# Supplementary material for: Improved transformation efficiency of group A Streptococcus by inactivation of a type I restriction modification system
Source: PLoS One. 2021 Apr 29;16(4):e0248201. doi: 10.1371/journal.pone.0248201 (PMC8084154; doi:10.1371/journal.pone.0248201)
Supplement: S1 Table — (DOCX) [file pone.0248201.s001.docx]

| **Table S1. Oligonucleotide primers used in this study** | | | |
| --- | --- | --- | --- |
| **Name** | **Description** | **Sequence** | **Description** |
| MAB38 | speB-qRT-PCR-F | 5'-TGCAGGTAGCTCTCGTGTTC | qRT-PCR analysis |
| MAB39 | speB-qRT-PCR-R | 5'-GCTTCCCAATCTTGTTTGCT | qRT-PCR analysis |
| MAB101 | hsdM screen F | 5'-GGGGCAATAATCGGAAGATG | hsdM sequencing |
| MAB102 | hsdM screen R | 5'-CGATTGGAACTACTTTATGGTTC | hsdM sequencing |
| MAB108 | hsdM mutant 5' R | 5'-CTTGCCTTCTGCAGCTCCAC-CAATTTCATTTAATGTTTTC | hsdM deletion construct |
| MAB109 | hsdM mutant 3' F | 5'-GAAAACATTAAATGAAATTG-GTGGAGCTGCAGAAGGCAAG | hsdM deletion construct |
| MAB185 | hsdM-R-SphI | 5'-CCCC-GCATGC-TTAGTTTCTAAAAGTATCCAATTCG | hsdM sequencing |
| MAB198 | emm1-qRT-PCR-F | 5'-CAAGTCGTCAAAGCCTTCGT | qRT-PCR analysis |
| MAB199 | emm1-qRT-PCR-R | 5'-GGCTTGCGTCTGAGATTTGT | qRT-PCR analysis |
| MAB200 | mga-qRT-PCR-F | 5'-CCAAGACCTTTCACGCTTGT | qRT-PCR analysis |
| MAB201 | mga-qRT-PCR-R | 5'-GGAGATGAACCCAGTTGGTC | qRT-PCR analysis |
| MAB202 | sclA-qRT-PCR-F | 5'-CACCTGTAGCCAACAACCAC | qRT-PCR analysis |
| MAB203 | sclA-qRT-PCR-R | 5'-CATCACTGCTACTGCTGCTG | qRT-PCR analysis |
| MAB204 | scpA-qRT-PCR-F | 5'-AGCCATATGCTGCGATCTCT | qRT-PCR analysis |
| MAB205 | scpA-qRT-PCR-R | 5'-GGGTTGAACCAAGTGTGCTT | qRT-PCR analysis |
| MAB206 | hsdM-qRT-PCR-F | 5'-TTGCCATCGAGAACCAACAC | qRT-PCR analysis |
| MAB207 | hsdM-qRT-PCR-R | 5'-GCAGTCGCTGACCATTTCAA | qRT-PCR analysis |
| MAB208 | hsdS-qRT-PCR-F | 5'-GCGTGTCTGGACAACACAAA | qRT-PCR analysis |
| MAB209 | hsdS-qRT-PCR-R | 5'-GCAACTCCACGCCCAATAAT | qRT-PCR analysis |
| MAB210 | hsdR-qRT-PCR-F | 5'-TTTGTTGTGGACGAATGCCA | qRT-PCR analysis |
| MAB211 | hsdR-qRT-PCR-R | 5'-CCGTATTGCTGTTCCGTTGT | qRT-PCR analysis |
| MAB226 | qRT-PCR grab-F | 5'-AAGCAGCAAGTTCAGATGCC | qRT-PCR analysis |
| MAB227 | qRT-PCR grab-R | 5'-AACAACCGCAGCTTCTTCTG | qRT-PCR analysis |
| MAB266 | hsdM_sequencing | 5'-GCTGATACCTTGGATGCAGAC | hsdM sequencing |
| MAB266 | hsdM_sequencing | 5'-GCTGATACCTTGGATGCAGAC | hsdM sequencing |
| MAB267 | hsd_sequencing_F1 | 5'-CCGCCACGGTTTGTGAGAG | hsd locus sequencing |
| MAB268 | hsd_sequencing_F2 | 5'-TCATGTCGAATGGGGATTAAAAAT | hsd locus sequencing |
| MAB269 | hsd_sequencing_F3 | 5'-GCATTGAAGCTTTTAACCAAGTC | hsd locus sequencing |
| MAB270 | hsd_sequencing_F4 | 5'-TCCTATGCGCAAAATGATATCTTT | hsd locus sequencing |
| MAB271 | hsd_sequencing_F5 | 5'-ACATATGTTAGCTGTCTTAGATAG | hsd locus sequencing |
| MAB272 | hsd_sequencing_F6 | 5'-CTCAGCACATTATTCAAGCTTTC | hsd locus sequencing |
| MAB273 | hsd_sequencing_F7 | 5'-ATGGATGATATCAACTATCACTATA | hsd locus sequencing |
| MAB274 | hsd_sequencing_F8 | 5'-GGAAAGGAAAGAGATGACAAAATC | hsd locus sequencing |
| MAB275 | hsd_sequencing_F9 | 5'-AAATGGTTGATCAATTGATTCAGTT | hsd locus sequencing |
| MAB276 | hsd_sequencing_F10 | 5'-AATGGTATCATTAAAATTGCTCAAG | hsd locus sequencing |
| MAB277 | hsd_sequencing_R | 5'-TTCAAAGGTCAGTGTCGGTTCG | hsd locus sequencing |
| MAB278 | M5005_Spy1634_qRTPCR_F | 5'-TAAAGCAGCGCAAGAAGGAG | qRT-PCR analysis |
| MAB279 | M5005_Spy1634_qRTPCR_R | 5'-TTGTCTGAGCATTGTGAGCA | qRT-PCR analysis |
| MAB280 | M5005_Spy1499_qRTPCR_F | 5'-AGCGTCGCTCTAGTGAAGAA | qRT-PCR analysis |
| MAB281 | M5005_Spy1499_qRTPCR_R | 5'-GTCAACCCTTCAACAGCCAA | qRT-PCR analysis |
| MAB282 | M5005_Spy1066_qRTPCR_F | 5'-CGCTGTCTTTAACCATGCCA | qRT-PCR analysis |
| MAB283 | M5005_Spy1066_qRTPCR_R | 5'-AGGTCCATGGATGGGTGTTC | qRT-PCR analysis |
| MAB284 | M5005_Spy1067_qRTPCR_F | 5'-TCTTGGCGAAATGGACAGAC | qRT-PCR analysis |
| MAB285 | M5005_Spy1067_qRTPCR_R | 5'-CTTGAGGCCAAGTGCCATAC | qRT-PCR analysis |
| MAB286 | M5005_Spy1762_qRTPCR_F | 5'-TGTCCTTGCAGGCACTCATA | qRT-PCR analysis |
| MAB287 | M5005_Spy1762_qRTPCR_R | 5'-AGGTAGCACGCTATCACCTG | qRT-PCR analysis |
| MAB288 | M5005_Spy1399_qRTPCR_F | 5'-GTTTGGCCTAGGCTTTACCG | qRT-PCR analysis |
| MAB289 | M5005_Spy1399_qRTPCR_R | 5'-CCAGATTTCAGCACGACCAG | qRT-PCR analysis |
| MAB290 | M5005_SpyT0008_qRTPCR_F | 5'-GCAGACGCACAGGACTTAAA | qRT-PCR analysis |
| MAB291 | M5005_SpyT0008_qRTPCR_R | 5'-CCGAGGACCGGAATCGAA | qRT-PCR analysis |
| MAB292 | M5005_SpyT0010_qRTPCR_F | 5'-GGGAAGTAGCTCAGCTTGGT | qRT-PCR analysis |
| MAB293 | M5005_SpyT0010_qRTPCR_R | 5'-TCGGGAAGACAGGATTCG | qRT-PCR analysis |
| MAB294 | M5005_Spy_1635_qRTPCR_F | 5'-AGCTGGAGCAGAAGCAGTTA | qRT-PCR analysis |
| MAB295 | M5005_Spy_1635_qRTPCR_R | 5'-AACTCAGGGCTTGCATTGTC | qRT-PCR analysis |
| MAB296 | M5005_Spy_1636_qRTPCR_F | 5'-TTGACGTAGTGACGCTCTCA | qRT-PCR analysis |
| MAB297 | M5005_Spy_1636_qRTPCR_R | 5'-CCAGAACAATCCAGAACCGT | qRT-PCR analysis |
| MAB298 | M5005_Spy_1637_qRTPCR_F | 5'-TCTGTGGTACTGGTGTTGGT | qRT-PCR analysis |
| MAB299 | M5005_Spy_1637_qRTPCR_R | 5'-GTATTCCGCGGCCAAGAAAG | qRT-PCR analysis |
| MAB300 | M5005_Spy_1638_qRTPCR_F | 5'-GCGCTCAGCATACATGACAA | qRT-PCR analysis |
| MAB301 | M5005_Spy_1638_qRTPCR_R | 5'-TTTGCAATTCCTGGAGCACT | qRT-PCR analysis |
| MAB302 | hsd_sequencing_R2 | 5'-AAAGATATCATTTTGCGCATAGGA | hsd locus sequencing |
| MAB303 | hsd_sequencing_R3 | 5'-GAAAGCTTGAATAATGTGCTGAG | hsd locus sequencing |
| MAB304 | hsd_sequencing_R4 | 5'-GATTTTGTCATCTCTTTCCTTTCC | hsd locus sequencing |
| MAB93 | hsdM mutant 5' F-BamHI | 5'-CCCC-GGATCC-GGTATCATTAAAATTGCTCAAGG | hsdM deletion construct |
| MAB96 | hsdM mutant 3' R-SalI | 5'-CCCC-GTCGAC-GAGAAATCATTGTAACTTCAAATAA | hsdM deletion construct |
| MW1 | recA-qRT-PCR-F | 5'-TGATTCTGGTGCGGTTTGATC | qRT-PCR analysis |
| MW2 | recA-qRT-PCR-R | 5'-ATTTACGCATGGCCTGACTC | qRT-PCR analysis |
| MW3 | hasB-qRT-PCR-F | 5'-TCCCCAAACGCTAATTGAAG | qRT-PCR analysis |
| MW4 | hasB-qRT-PCR-R | 5'-TTAAACGGTAAACCCCGACT | qRT-PCR analysis |
| MW5 | slo-qRT-PCR-F | 5'-GCCCTTCAGCTGGCTAATAA | qRT-PCR analysis |
| MW6 | slo-qRT-PCR-R | 5'-TTGTCTCCCATACCTGGTAAATC | qRT-PCR analysis |
| MW7 | nga-qRT-PCR-F | 5'-GCGTCACGTGCTGAGTATTA | qRT-PCR analysis |
| MW8 | nga-qRT-PCR-R | 5'-AAGCTCCGCTTTCTTTGTAGA | qRT-PCR analysis |
